# Mitral annular plane systolic excursion (MAPSE) in shock: a valuable echocardiographic parameter in intensive care patients

**DOI:** 10.1186/1476-7120-11-16

**Published:** 2013-05-30

**Authors:** Lill Bergenzaun, Hans Öhlin, Petri Gudmundsson, Ronnie Willenheimer, Michelle S Chew

**Affiliations:** 1 Department of Anaesthesiology and Intensive Care, Skåne University Hospital, Institute for Clinical Sciences Malmö, Lund University, Malmö, S-20502, Sweden; 2 Department of Cardiology, Skåne University Hospital, Lund University, S- 22185 Lund, Sweden; 3 Department of Biomedical Science, Malmö University, Malmö, S- 20506, Sweden; 4 Heart Health Group, Lund University, Geijersg. 4C, Limhamn, 21618, Sweden; 5 Department of Anesthesia and Intensive Care, Hallands Hospital Halmstad and Institute for Clinical Sciences Malmö, Halmstad, S-30185, Sweden

**Keywords:** Echocardiography, Intensive care, Mitral annular plane systolic excursion, Outcome

## Abstract

**Background:**

Assessing left ventricular (LV) dysfunction by echocardiography in ICU patients is common. The aim of this study was to investigate mitral annular plane systolic excursion (MAPSE) in critically ill patients with shock and its relation to LV systolic and diastolic function, myocardial injury and to outcome.

**Methods:**

In a prospective, observational, cohort study we enrolled 50 patients with SIRS and shock despite fluid resuscitation. Transthoracic echocardiography (TTE) measuring LV function was performed within 12 hours after admission and daily for a 7-day observation period. TTE and laboratory measurements were related to 28-day mortality.

**Results:**

MAPSE on day 1 correlated significantly with LV ejection fraction (LVEF), tissue Doppler indices of LV diastolic function (é, E/é) and high-sensitive troponin T (hsTNT) (p< 0.001, p= 0.039, p= 0.009, p= 0.003 respectively) whereas LVEF did not correlate significantly with any marker of LV diastolic function or myocardial injury. Compared to survivors, non-survivors had a significantly lower MAPSE (8 [IQR 7.5-11] versus 11 [IQR 8.9-13] mm; p= 0.028). Other univariate predictors were age (p=0.033), hsTNT (p=0.014) and Sequential Organ Failure Assessment (SOFA) scores (p=0.007). By multivariate analysis MAPSE (OR 0.6 (95% CI 0.5- 0.9), p= 0.015) and SOFA score (OR 1.6 (95% CI 1.1- 2.3), p= 0.018) were identified as independent predictors of mortality. Daily measurements showed that MAPSE, as sole echocardiographic marker, was significantly lower in most days in non-survivors (p<0.05 at day 1–2, 4–6).

**Conclusions:**

MAPSE seemed to reflect LV systolic and diastolic function as well as myocardial injury in critically ill patients with shock. The combination of MAPSE and SOFA added to the predictive value for 28-day mortality.

## Background

In intensive care patients a frequently applied method for estimating LV systolic function is left ventricular ejection fraction (LVEF) [1]. LV systolic dysfunction has been observed in critically ill patients with shock [2,3] although there is conflicting evidence that LV systolic impairment is associated with mortality [2,4,5].

Mitral annular plane systolic excursion (MAPSE) also known as left atrioventricular plane displacement (AVPD), mitral annulus excursion (MAE) or mitral ring displacement is an M-mode derived echocardiographic marker of LV longitudinal function [6-8]. MAPSE correlates well with other markers of LV function [6,9,10], is easily obtainable [11-13] even for the untrained observer [12] and in patients with poor acoustic windows [8]. It has been suggested as a surrogate measurement for LVEF in cardiac patients [12,14]. A reduced MAPSE has been shown to correlate with age, and LV function in patients with myocardial infarction, heart failure and atrial fibrillation [15-17] and to be more sensitive than conventional echocardiographic markers in detecting abnormalities in LV systolic function at an early stage [7,18]. MAPSE is known to be prognostic for major cardiac events and mortality in patients with cardiovascular disease [15,19-21]. In critically ill patients there are no reports of the use of MAPSE, its association with other echocardiographic parameters, myocardial injury or clinical outcome.

The aim of this study was to investigate if MAPSE is of prognostic significance in critically ill patients with shock. Further, we wanted to examine if MAPSE correlates with other markers of LV function and myocardial injury.

## Material and methods

The study was approved by the Regional Ethics Review Board, Lund, Sweden (Dnr.187/2005). Informed consent was sought from the patient or, if not possible, from the next of kin. The study design was a prospective observational cohort study. Patients >18 years old admitted to the mixed-bed ICU of Skåne University Hospital, Malmö, Sweden, were screened for eligibility. We included 55 consecutive patients with Systemic Inflammatory Response Syndrome (SIRS) and concurrent shock, where shock was defined as failure to maintain mean arterial pressure ≥ 70 mmHg, despite adequate fluid resuscitation according to the surviving sepsis campaign algorithm [22]. Exclusion criteria were pregnancy, known abnormalities of coagulation, fibrinolytic therapy, compromised immunity or a “Do Not Attempt Resuscitation” order. Acute Physiology and Chronic Health Evaluation (APACHE) II scores [23] were calculated at admission and Sequential Organ Failure Assessment (SOFA) scores [24] were calculated daily. After the initial resuscitation period, fluids were given at the treating clinician’s discretion. Mean arterial pressure (MAP), heart rate (HR), positive end expiratory pressure (PEEP) and vasopressor (norepinephrine) dose was recorded at the time of the TTE examination. Blood samples were taken from an indwelling arterial line within 12 h of inclusion. High-sensitive troponin T (hsTNT) and B-natriuretic peptide (BNP) were analyzed as reported previously [25]. Patients were followed for 7 days or until discharge from ICU. Mortality was defined as 28-day all cause mortality.

### Transthoracic echocardiography

Transthoracic echochardiography (TTE) was performed within 12 hours after admission and daily for a 7-day observation period or until discharge from ICU by one of four experienced echocardiographers (LB, MC, PG, MD). Images were acquired using a Hewlett- Packard Sonos 5500 (Andover, Mass, U.S.A) scanner and a 3 MHz transducer. Two-dimensional (2D) imaging examinations were performed in the standard apical four- and two- chamber views (2C- and 4C views). Tissue harmonic imaging was used to enhance 2D image quality. LV ejection fraction (LVEF) was assessed by visual estimation of EF, based on “eyeball” ejection fraction. M-mode images were obtained at the LV septal, lateral, anterior, and posterior borders of the mitral ring [18] in the apical 2C- and 4C views, and an average mitral annular plane systolic excursion (MAPSE) value was calculated. Pulsed-wave (PW) tissue Doppler recorded the peak systolic velocity (TDIs) of the LV septal wall at the level of the mitral annulus in the apical 4C view [26]. Transmitral velocities were measured with PW Doppler in the 4C view. For LV diastolic function, we used the mitral inflow profile, the E- and A-velocity and calculated the E/A ratio. PW tissue Doppler recorded the diastolic velocities (é) of the LV septal wall at the level of the mitral annulus in the apical 4C view. The E/é ratio, an index of LV filling pressure, was calculated and é (septal) < 8cm/s indicated diastolic dysfunction [27]. All TTE studies were recorded over three consecutive cardiac cycles independently of the respiratory cycle and averaged. In patients with non-sinus rhythm measurements were collected over 5–10 heartbeats. Analyses of the measurements were made in Phillips digital storing and analysis software Xcelera (Best, the Netherlands) offline.

### Statistical analysis

Data are presented as median (lower quartile: upper quartile), percentages or absolute values. For not normally distributed variables we used non-parametric test exclusively. For correlation between two variables, Spearman’s rank correlation was used and for differences between two groups we used Mann-Whitney’s U-test. Categorical data were analyzed with Fisher’s exact test. HsTNT and BNP were log transformed with natural logarithm due to skewed distribution. Discrimination analysis was performed using receiver operating characteristics (ROC) curve under the area using multiple logistic regression predictions. Our aim was to investigate how 28-day mortality can be predicted by more than one explanatory variable measured early during ICU stay. Since we did not have any censored data during this period and odds ratio was the outcome of interest, logistic regression was chosen to be the most suitable method [28]. Multivariate (backward stepwise selection method with probability for the removal of 0.10) logistic regression analyses were used to determine the association of variables with 28-day mortality. The Hosmer and Lemeshow test of goodness of fit was used to indicate if the model provides an adequate fit to the data. Odds ratios (OR) were calculated. We have used the method described by Hoaglin and Iglewicz [29] to test for outliers. The intra- and inter-observer variability of echocardiographic parameters was measured by the coefficient of variation (CV). CV was defined as the ratio of the standard deviation to the mean multiplied by 100. All probability values are two-tailed and significance was set at p < 0.05. The analyses were performed using SPSS 18.0 (SPSS, Chicago, IL, U.S.).

## Results

### Patient characteristics

The original study included 55 consecutive patients. Two patients were excluded due to lack of written consent. In two patients TTE examinations were not recorded (death before possible TTE and morbid obesity respectively) and one patient was incorrectly registered in the echocardiography database. These five patients were excluded resulting in a total of 50 analyzed patients. Of 350 expected echocardiographic examinations, 96 were missing because of death or discharge from the ICU before Day 7. Another 23 examinations were lost during the installation of a new offline storing and analysis system. Thus, in total, 231 examinations were available for analysis. An additional file shows this in more detail see (Additional file 1). Two-thirds of the population suffered from septic shock and one-third from shock due to other causes (pancreatitis, post-major non-cardiac surgery, intoxication and multiorgan failure, gastrointestinal bleeding and portal hypertension or unknown cause). Pre-existing cardiac disease was present in 12 (24%) patients defined as severe arrhythmia, heart failure or ischemic heart disease. Forty-five (90%) patients were mechanically ventilated at inclusion. Acute kidney failure was present in 14 (28%) patients requiring continuous renal replacement therapy (CRRT), including one patient with chronic kidney failure (Table 1). Twelve patients (24%) received dobutamine and one (2%) adrenaline at inclusion. The median ICU length of stay was 8 (IQR 4–13) days. Eight patients died within seven days and thirteen patients after 28 days with an all cause 28-day mortality of 26%.

**Table 1:** Baseline and echocardiographic characteristics of studied patients at day 1

	**All n=50**	**Survivors n=37**	**Non-survivors n=13**	**p**
**Clincal data**				
Median age, y	65 (54–74)	60.5 (49.5–72.0)	72.0 (69.0–76.0)	0.033
Female sex, n (%)	14 (28)	12 (32)	2 (15)	>0.1
Diabetes mellitus, n (%)	6 (12)	5 (14)	1 (8)	>0.1
Hypertension, n (%)	12 (24)	11 (30)	1 (8)	>0.1
Cardiac disease, n (%)	12 (24)	8 (22)	4 (31)	>0.1
APACHE II score	24 (19–29)	24 (17–28)	28 (21–34)	0.060
SOFA score	12 (9–14)	10 (9–13)	13 (13–14)	0.007
HR, beats/min	99 (85–115)	99 (85–116)	98 (83–107)	>0.1
MAP, mmHg	76 (70–88)	76 (70–90)	76 (73–79)	>0.1
NE dose, μg/kg/min	0.09 (0.05-0.14)	0.08 (0.04-0.13)	0.1 (0.09-0.18)	>0.1
CRRT, n (%)	14 (28)	11 (30)	3 (23)	>0.1
Fluids, ml/kg/24h	43 (22–84)	38 (20–81)	46 (28–77)	>0.1
Mechanical ventilation, n (%)	45 (90)	35 (95)	10 (77)	>0.1
Peep, cmH2O	8 (5–10)	8 (5–10)	10 (9–13)	0.083
**Biochemical data**				
Creatinine, μmol/L	156 (94–243)	138 (86–221)	182 (118–246)	>0.1
Lactate mmol/L	2.2 (1.6-3.2)	2.2 (1.6-3.2)	2.6 (1.8-4.6)	>0.1
hsTNT, ng/L	79.5 (23–182)	77.5 (18.6-125.3)	152 (80–501)	0.014
BNP, pmol/L	187 (98–375)	157 (84–356)	262 (183–440)	>0.1
**Echocardiographic data**	n=47	n=34	n=13	
LVEF, %	49 (40:55)	50 (40–50)	45 (35–65)	>0.1
LVEF, %, mean (±SD)	47 (±14.8)	47 (±12.8)	48 (±19.7)	
MAPSE, mm	10 (8.0-12.6)	11 (8.9-13)	8 (7.5-11)	0.028
MAPSE, mm, mean (±SD)	10.3 (±2.8)	10.9 (±2.9)	8.7 (±2.5)	
TDIs cm/s	8.9 (7.1-10.0)	8.9 (7.2-9.9)	7.5 (6.6-10)	>0.1
E/A	1.2 (0.9-1.5)	1.2 (0.9-1.5)	1.3 (0.9-1.6)	>0.1
é, cm/s	8.3 (7–10)	8.4 (7.5-10.5)	7.2 (6.3-8.6)	>0.1
é, cm/s, mean (±SD)	8.7 (±2.2)	8.9 (±2.0)	8.0 (±2.6)	
E/é	10.2 (8.5–12)	10.0 (8.2–11.5)	13.0 (9.6–15.2)	0.055
E/é, mean (±SD)	10.7 (±3.7)	9.8 (±2.8)	12.7 (±5.0)	

Data are presented as median (lower quartile- upper quartile), as mean (± SD) or in numbers (%). APACHE II (Acute Physiology and Chronic Health Evaluation), CRRT (Continuous Renal Replacement Therapy), SOFA (Sequential Organ Failure Assessment), HR (heart rate), MAP (mean arterial pressure), NE (norepinephrine), Peep (positive end expiratory pressure).

### Mitral annular plane systolic excursion (MAPSE) and relation to other echocardiographic parameters

On day 1 a total of 47 echocardiographic examinations were available for analysis, since 3 examinations were lost during the installation of a new offline storing and analysis system. Results on day 1 showed that MAPSE was significantly lower in non-survivors (median 8 [IQR 7.5-11] mm) than in survivors (median 11 [IQR 8.9-13] mm) of 28-day mortality (p= 0.028). No other echocardiograohic parameter showed any significant difference (Table 1). In 14 (30%) patients ejection fraction was preserved (LVEF ≥ 55%) and in 33 patients (70%) impaired (LVEF ≤ 50%) with no significant difference in mortality between these two groups. Six patients had severely reduced LV systolic function (LVEF < 30%). MAPSE was 11 [11–12.8] mm in patients with preserved EF and 9 [7.3-12.3] mm in those with reduced EF (p= 0.069). MAPSE correlated significantly with LVEF, é, E/é and hsTnT whereas LVEF did not correlate significantly with markers of LV diastolic function, filling pressure or cardiac biomarkers (Table 2). MAPSE showed a negative correlation with age (r=−0.411, p=0.003) but was not associated with previous hypertension or cardiac disease. LV diastolic dysfunction (é < 8 cm/s) showed a significant negative association with MAPSE (p= 0.047) whereas there was none with LVEF.

**Table 2:** Correlation (r) between markers of LV systolic function with LV diastolic function and cardiac biomarkers

	**MAPSE**		**TDIs**		**LVEF**	
	**r**	**p**	**r**	**p**	**r**	**p**
*LV systolic function*						
LVEF	0.594	<0.001	0.649	<0.001		
*LV diastolic function*						
é	0.309	0.039	0.341	0.022		ns
E/é	-0.383	0.009		ns		ns
*Cardiac biomarkers*						
hsTNT	-0.428	0.003		ns		ns
BNP		ns		ns		ns

Spearman rank correlation was used. Statistical significance at p< 0.05.

The intra- and inter-observer variability for MAPSE was 4.4% and 5.3% respectively, and for other echocardiographic markers as reported previously [13,25].

### Mortality

**Figure 1:**
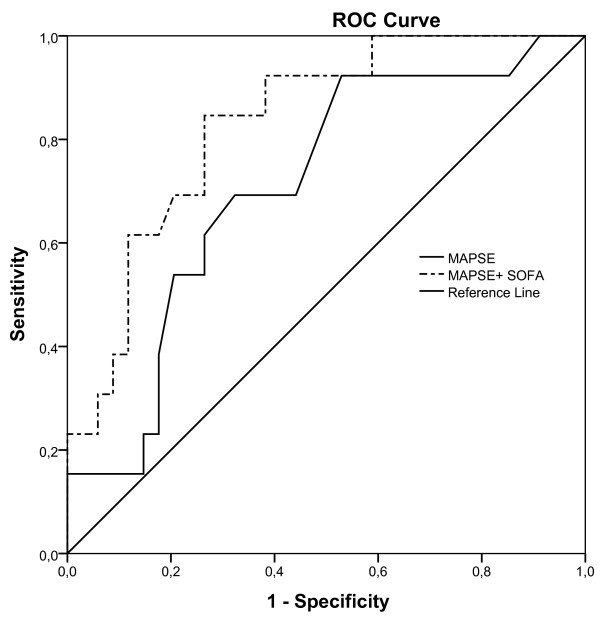
**Receiver operating characteristic (ROC) for MAPSE and for a combined predictor consisting of MAPSE and SOFA score.** With regards to 28-day mortality the area under the curve (AUC) for MAPSE was 0.709 (95% CI 0.548- 0.870, p= 0.028) and for the combined predictor 0.831 (95% CI 0.711- 0.952, p<0.001).

MAPSE (p= 0.028), SOFA score (p= 0.007), age (p= 0.033) and hsTNT (p= 0.014) were identified as univariate predictors of 28-day mortality (Table 3). A multivariate logistic regression analysis including these variables identified a model with MAPSE (p= 0.015) and SOFA (p= 0.018) as independent predictors of 28-day mortality. The Hosmer and Lemeshow test for the model was non-significant (p=0.998) indicating adequate fit to the data. The adjusted OR for MAPSE and SOFA score were 0.6 (95% CI 0.5- 0.9) and 1.6 (95% CI 1.1- 2.3) respectively (Table 3). With regards to 28-day mortality the area under the curve (AUC) at day 1 for MAPSE was 0.709 (95% CI 0.548- 0.870, p = 0.028) with 69% sensitivity and 68% specificity for a cut-off value of 8 mm and AUC for SOFA score was 0.733 (95% CI 0.592- 0.874, p = 0.014) with 69% sensitivity and 68% specificity for a cut-off value of 12. When adding MAPSE to SOFA score, the AUC for the combined predictor increased to 0.831 (95% CI 0.711- 0.952, p <0.001) (Figure 1).

**Table 3:** Multivariate analysis for predictors of death in patients with shock

	**Multivariate analysis**		
	**Odds Ratio (95%CI)**	**Wald statistics**	**p**
MAPSE (mm)	0.666 (0.5-0.9)	5.915	0.015
SOFA score	1.596 (1.1-2.3)	5.638	0.018

### Cardiac biomarkers

Results at day 1 showed that hsTNT was significantly higher in non-survivors (152 [IQR 80–501] ng/L) than in survivors (77.5 [IQR 18.6-125.3] ng/L) (p= 0.014) whereas there was no difference in BNP (Table 1). In patients with diastolic dysfunction (é< 8cm/s) both hsTNT and BNP were significantly higher in non-survivors compared to survivors (p= 0.020 and p= 0.039 respectively); this was not seen in those with systolic dysfunction (LVEF≤ 50%). hsTNT showed a significant negative correlation with MAPSE but not with LVEF or TDIs (Table 3). In patients with diastolic dysfunction (é< 8cm/s) hsTNT and BNP showed a significant negative correlation with MAPSE (r= −0.478, p= 0.033; borderline for BNP r= −0.441, p= 0.051) but there was none with TDIs.

**Figure 2:**
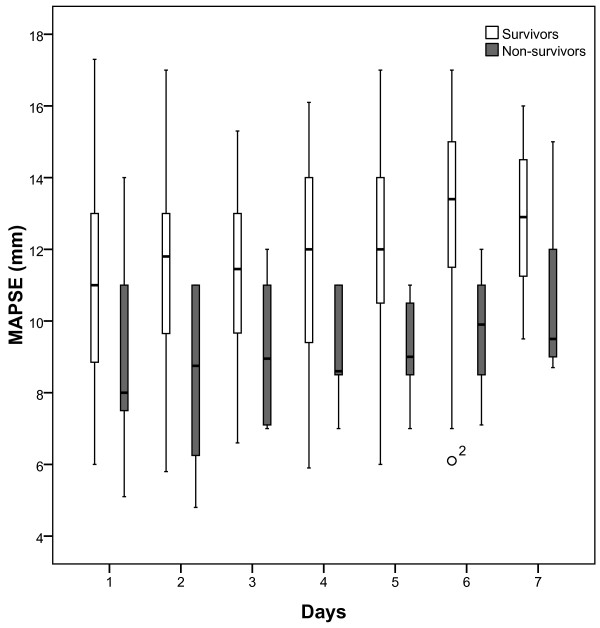
**Boxplots of daily measurements show that MAPSE is significantly lower in non-survivors (grey) of 28-day mortality compared to survivors (white) in most days (day 1 p=0.028, day 2 p=0.003, day 3 p=0.060, day 4 p= 0.036, day 5 p=0.026, day 6 p=0.017, day 7 p=0.075).** ° Value and case number within 1.5 boxplot length (SPSS 18.0).

**Figure 3:**
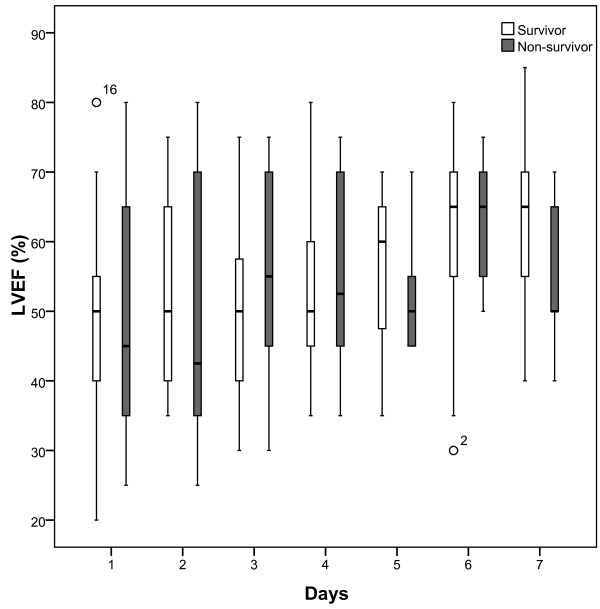
**Boxplots of daily measurements show that there is no significant difference (p>0.1) at any day in LVEF between survivors (white) and non-survivors (grey) of 28-day mortality.** ° Value and case number within 1.5 boxplot length (SPSS 18.0).

Daily measurements over 7 days showed that in non-survivors MAPSE was significantly lower in most days (day 1 p=0.028, day 2 p=0.003, day 3 p=0.060, day 4 p= 0.036, day 5 p=0.026, day 6 p=0.017, day 7 p=0.075) (Figure 2) whereas LVEF was not significant different (p>0.1) at any day (Figure 3).

## Discussion

Our main findings are that MAPSE was an independent predictor of 28-day mortality in critically ill patients with shock and systemic inflammation. Combining MAPSE with SOFA increased the predictive value for mortality. MAPSE correlated with markers of LV systolic and diastolic function as well as myocardial injury, whereas LVEF did not.

### MAPSE and prognosis

In critically ill patients echocardiography has gained popularity as a tool for assessing LV function [5,30]. LVEF is probably the most commonly used and accepted method of measuring LV systolic function in this setting [1] however its usefulness in predicting mortality has produced conflicting results [2,5,31]. In patients with septic shock studies measuring the LV longitudinal function by tissue Doppler imaging (TDI) have moved into focus during the recent years identifying mainly diastolic TDI indices as prognostic markers whereas systolic TDI parameters seem to be less consistently related to mortality [31-34]. Interestingly in none of these studies MAPSE was used as a marker of LV systolic function.

MAPSE is a simple, easily obtained parameter and may contribute to the evaluation of systolic function. MAPSE was obtainable in all patients, and showed inter- and intra-observer variability of 4.4% and 5.3% [13]. It is less well investigated than its right ventricular counterpart, tricuspid annular plane systolic excursion (TAPSE), and has received considerably less attention than TDI variables. In critical care settings where acoustic windows are often suboptimal, MAPSE seems to be an attractive parameter. A decreased MAPSE is known to be associated with conditions affecting LV function such as myocardial infarction, heart failure, atrial fibrillation and age [15-17] and its relation to mortality has been described by several studies in patients with cardiovascular disease [15,19-21].

We found that MAPSE on day 1 was significantly lower in non-survivors compared to survivors and could together with SOFA score be identified as independent predictors of 28-day mortality. Further, combining MAPSE and SOFA score seemed to increase the risk of death. These results are strengthened by the finding that MAPSE was significantly decreased in non-survivors compared to survivors in most days of the 7-day observation whereas LVEF was not. LVEF, on the other hand, being near normal could not be identified as a prognostic marker and we speculate if this, like in patients with cardiovascular disease and preserved ejection fraction, could be due to LVEF being less sensitive in uncovering subtle myocardial changes [35,36].

### MAPSE in relation to other markers of LV function and myocardial injury

Previous studies in patients with cardiovascular disease have suggested MAPSE as a surrogate measurement for LVEF with both normal and reduced LV function [12,14]. A mean value for MAPSE of > 10 mm was linked with preserved EF (≥ 55%) and values < 8 mm with reduced EF (< 50%) [6,19,37]. Our results are in line with this, with MAPSE correlating significantly with LVEF. MAPSE was 11 [11–12.8] mm in patients preserved EF and in those with reduced EF slightly higher than 8 mm (MAPSE 9 [7.3-12.3] mm).

Although MAPSE and LVEF may be related, they are not entirely interchangeable [13,17]. MAPSE is suggested to be primarily representative of subendocardial, longitudinally oriented, myocardial fibres compared to the subepicardial, circumferential fibres measured by LVEF, and is known to detect more subtle abnormalities of LV function [7,18]. This is seen in patients with increasing age, myocardial hypertrophy or diastolic dysfunction with preserved ejection fraction (HFpEF) where long axis function of the heart is already impaired while the radial function can be preserved or even increased [18,35]. Thus by using LVEF the long axis function of the heart is not necessarily considered.

Similar to MAPSE, tissue Doppler imaging is described to be superior to conventional echocardiography in detecting abnormalities of LV function [38] and its correlation with MAPSE has been described previously [39]. In a recent study (Wenzelburger), TDI indices of both LV diastolic and systolic function correlated significantly with MAPSE in patients with HFpEF [35] illustrating the close relationship between systolic and diastolic LV function. LV systolic torsional (twisting) deformation is one mechanism by which potential energy is stored. After systole the heart relaxes or untwists, an energy releasing process, and aids to early LV filling by suctioning [40]. Thus a decrease in atrioventricualar plane motion will result in less energy stored during systole and hence reduced LV diastolic mechanics.

The relationship between MAPSE and TDI indices is supported by our results where MAPSE correlated significantly with é, a diastolic marker, and showed a negative significant correlation with E/é, a surrogate marker for LV filling pressure. Additionally we found a significant association between diastolic dysfunction (é< 8cm/s) and MAPSE, but none with LVEF. Of note, in our previous study we showed a significant correlation between MAPSE and the systolic marker of tissue Doppler imaging (TDIs) (r= 0.427, p< 0.01) [13].

Notably, MAPSE and TDI are not interchangeable. The two parameters describe different vector components of longitudinal systolic motion. Although TDIs has been described to correlate well with MAPSE in studies with healthy individuals [39,41], they differ in some important aspects [42] as MAPSE measures the entire systolic phase including isovolumetric contraction [15] in contrast to TDIs. In addition a blurred TDI signal can be a confounding factor when measuring the velocity [43] and in these situations MAPSE can be a valuable. Overall we believe that MAPSE and tissue Doppler measurements are important echocardiographic parameters in assessing LV function.

Finally, we sought to investigate if there was a relationship between cardiac biomarkers (hsTNT and BNP) and MAPSE and found that hsTNT but not BNP was significantly higher in non-survivors and correlated significantly with MAPSE but not LVEF. This is in line with a recent study in septic neonates were hsTNT was significantly higher in non-survivors and correlated with longitudinal LV systolic function measured by TDI but not with fractional shortening [44]. Landesberg et al. [32] found that hsTNT was significantly higher in patients with decreased é and LVEF. This is similar to our findings where hsTNT in patients with diastolic dysfunction showed a significant negative correlation with MAPSE. Although we found no relationship between LVEF and cardiac troponin T our findings are generally in support of these studies and we speculate if MAPSE may be more sensitive than LVEF in detecting early myocardial changes in critically ill patients with shock.

### Limitations

Firstly, we used eyeballing to measure LVEF. Simpson’s biplane method is the currently accepted standard. We and others have previously shown that eyeball EF was as good as the Simpson’s method [13,45], and was more easily obtained in ICU patients [13]. Although unlikely, we cannot exclude that using Simpson’s method for measuring LVEF could have influenced our results. Secondary analysis (data not shown) in 44 patients where good-quality Simpson’s EF could be obtained showed no relationship to mortality. Secondly, we did not screen our patients for specific conditions affecting MAPSE measurements such as localized wall motion abnormalities or mitral annular calcifications. Thirdly, no dynamic fluid responsiveness tests were used limiting our results, as MAPSE in analogy with tissue Doppler measurements, is affected by changing fluid conditions. Finally, the multivariate analysis was limited by the small number of patients in this study, and we cannot exclude other confounding factors. Nevertheless, we clearly demonstrate a relationship between MAPSE and 28-day mortality.

## Conclusions

In this study we showed that MAPSE but not LVEF correlated with other markers of LV function and myocardial injury and could be identified as an independent predictor of 28-day mortality. Its predictive value increased when added to SOFA score. A reduced MAPSE persisted in non-survivors throughout the ICU stay. Future prospective studies should evaluate the advantages and weaknesses of MAPSE in critically ill patients with shock where vasoactive drugs and positive pressure ventilation are commonly used, all influencing echocardiographic measurements.

## Competing interests

The authors declare that they have no competing interests.

## Authors’ contributions

LB, RW, MC, PG have made substantial contributions to conception and design of the study. LB, MC and HO participated in interpretation of data, helped to draft the manuscript. PG and LB made substantial contributions in acquisition and analysis of data. All authors have made substantial intellectual contributions to the manuscript and have given final approval of the version to be published.

## Supplementary Material

Additional file 1 Flow diagram showing number of patients, echocardiographic examinations and hsTNT measurements. Click here for file
