# Supplementary figures and images for: Mitral annular plane systolic excursion (MAPSE) in shock: a valuable echocardiographic parameter in intensive care patients
Source: Cardiovasc Ultrasound. 2013 May 30;11:16. doi: 10.1186/1476-7120-11-16 (PMC3679845; doi:10.1186/1476-7120-11-16)

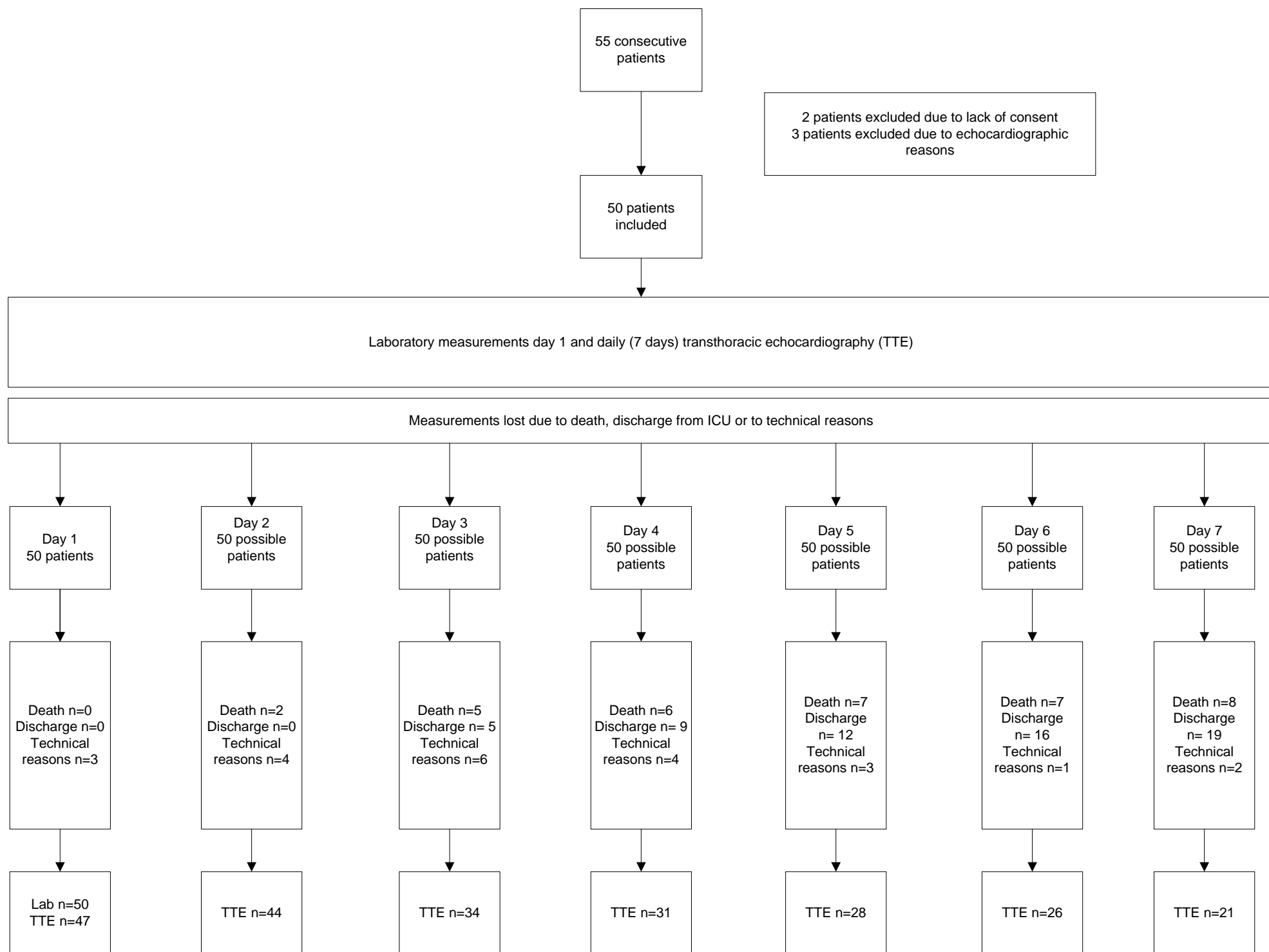

Supplement: Additional file 1 — Flow diagram showing number of patients, echocardiographic examinations and hsTNT measurements. [file 1476-7120-11-16-S1.pdf]
